# Short-Term Cigarette Smoking in Rats Impairs Physical Capacity and Induces Cardiac Remodeling

**DOI:** 10.1155/2020/2589892

**Published:** 2020-11-23

**Authors:** Danilo Sales Bocalini, Rafael da Silva Luiz, Kleiton Augusto Santos Silva, Andrey Jorge Serra, Renata Andrade Avila, André Soares Leopoldo, Ana Paula Lima-Leopoldo, Márcia Regina Holanda da Cunha, Paulo Jose F. Tucci, Leonardo dos Santos

**Affiliations:** ^1^Experimental Physiology and Biochemistry Laboratory, Center of Physical Education and Sports, Federal University of Espírito Santo, Vitoria, Brazil; ^2^Department of Medicine, Nephrology Division, Federal University of São Paulo, São Paulo, Brazil; ^3^Department of Biomedical Sciences, University of Missouri, Columbia, MO, USA; ^4^Department of Medicine, Cardiology Division, Federal University of São Paulo, São Paulo, Brazil; ^5^Department of Physiological Sciences, Center of Health Sciences, Federal University of Espirito Santo, Vitoria, Brazil

## Abstract

Despite the strong evidence on the cardiac and renal damages after chronic exposure to cigarette smoke, there is a paucity of data on its short-term effects. The study evaluated the short-term effects of cigarette smoking on left ventricular (LV) remodeling, *in vitro* myocardial and renal function. Female *Wistar* rats were randomized to control (C) and cigarette smoking rats for eight weeks. Physical capacity was assessed using an adapted model of exhaustive swim; left ventricle (LV) morphology and function were also evaluated. Renal function was assessed by creatinine clearance and urine protein. The *in vitro* myocardial performance was analyzed in isolated papillary muscles. Rats exhibited reduced physical capacity after short-term cigarette smoking. Although there was no change on LV function, reduced chamber diameter was found in the smoking group associated with an increased LV wall thickness. There was augmented cardiac mass compared to C that was confirmed by increased cardiomyocyte nucleus volume, but *in vitro* myocardial performance and renal function were unchanged. A short-term cigarette smoking induces cardiac remodeling without abnormalities in function. The smoking group still preserved renal function and *in vitro* myocardial performance. However, the reduced physical capacity may suggest an impairment of the cardiac reserve.

## 1. Introduction

Cigarette smoking is the leading cause of preventable morbidity and premature mortality at developed countries [1]. The main health disorders linked to smoking are cardiovascular diseases, cancer, and chronic obstructive pulmonary disease. In addition, smoking is an important risk factor to myocardial infarction [2] and nephropathies [3].

Studies have reported that a long-term cigarette smoke exposure may be associated with cardiac remodeling [4–6] which in turn correlates with myocardial dysfunction, heart failure, and increased mortality risk [7]. However, there are few studies [8, 9] that define the cardiotoxic effects of short-term cigarette use, specifically reporting data from myocardial mechanics, which could indicate the initial and possibly reversible changes in the cardiocirculatory system. If concerning renal implications, although long-term exposure to cigarette smoke is an independent risk factor for microalbuminuria, diuresis and proteinuria [10, 11], the short-term effects on the renal function are still poorly understood as well.

Thus, to date, a number of evidences have been reported the local and systemic effects of chronic exposure to cigarette smoke and such studies requiring prolonged exposure for several months. Therefore, the aim of this study was to evaluate the effects of short-term cigarette smoke exposure on myocardial function and remodeling and renal function and its consequences to the physical capacity of previously healthy rats.

## 2. Materials and Methods

Twenty female *Wistar* rats, weighing 200–230 g, were assigned to one of the following two groups: smoking animals (*n* = 10), exposed to cigarette smoke for eight weeks, and control animals (*n* = 10), allocated to the similar chamber but not exposed to tobacco smoke. The environment was controlled in terms of light (12 h light/dark cycle), clean-air room temperature (23 ± 3°C), and relative humidity (60 ± 5%). The study was conducted in accordance with the Basic & Clinical Pharmacology & Toxicology policy for experimental and clinical studies [12]. All procedures performed were in accordance with the Guide for the Care and Use of Laboratory Animals (NIH) and the ethical standards of the institution (Institutional Research Ethics Committee of the Federal University of São Paulo—protocol 34/08).

### 2.1. Exposure to Tobacco Smoke

Rats were exposed to cigarette smoke in a chamber (dimension: 1.000 × 800 × 700 mm) connected to a smoking device according to other studies [5, 6]. The smoke was drawn out of filtered commercial cigarettes (composition per unit: 1.1 mg nicotine, 14 mg tar, and 15 mg carbon monoxide) with a vacuum pump and exhausted into the smoking chamber. In the first week, cigarette number was gradually increased from 5 to 20 cigarettes for 60 min, administered two times a day. Subsequently, 20 cigarettes were used in each smoking session, twice a day (60 min in the morning and 60 min in the afternoon) for eight weeks. The chamber carbon monoxide content was measured with a sensor Toxi Vision CO 860 (Biosystems, Prairieville, LA, USA).

### 2.2. Physical Capacity Swim Test

Physical capacity was assessed using an adapted model of exhaustive swimming previously reported [13]. A load equivalent to 10% of body weight was attached around the waist of each rat using a rubber band. Rats from all groups were observed to determine swimming time until exhaustion. Exhaustion was defined as the time-point when the rat could not swim up to the water surface for 10 seconds.

### 2.3. Renal Function

Renal function was measured after 8 wk cigarette smoking exposure as previously described [14]. Briefly, rats were placed in a metabolic cage for 24 h with water and food *ad libitum*. After urine collection, samples were stored in −20°C. Serum (SCr, mg/dL) and urinary creatinine (UCr, mg/dL) were determined by Jaffé method (Creatinina K-Colorimetrico, Picratoalcalino, Labtest Diagnostica SA, Minas Gerais, Brazil). The creatinine clearance (CrCl, mL/min) was calculated to estimate glomerular filtration rate. The urinary protein was measured by an enzymatic colorimetric assay (Sesiprot kit Labtest Diagnostica SA, Minas Gerais, Brazil) and expressed as 24-hour proteinuria (24 h Uprot) or urine protein/creatinine clearance ratio (Uprot/CrCl).

### 2.4. Blood Pressure Measurement

Animals were allowed to adapt to the environment for three days before the measurement of blood pressure. Animals were placed in a heated chamber at a temperature of 38-40°C for ten minutes, and blood pressure values were recorded from each animal. At the end of protocols, mean, and systolic and diastolic blood pressures were determined by a tail cuff method (LETICA, LE5002, Barcelona, Spain) in conscious rats.

### 2.5. Doppler Echocardiography

At the end of protocols, rats were anesthetized with ketamine (50 mg/kg) plus xylazine (10 mg/kg) by i.p. route. Transthoracic echocardiographic was performed using a HP SONOS 5500 instrument (Philips Medical Systems, Andover, MA, USA), as described elsewhere [15, 16]. The transverse images were obtained at basal (at the tip of the mitral valve leaflets), middle (at the papillary muscle level), and apical (distal to the papillary muscle but beyond the cavity cap) levels of the left ventricular (LV). The end-diastolic (LVEDD) and systolic (LVESD) LV diameters and diastolic (LVAWT) and systolic (LVPWT) LV posterior wall thicknesses were measured from the transverse parasternal view using M-mode images. The LV systolic function was defined by the fractional shortening (FS), and diastolic function was analyzed by the mitral diastolic influx velocity curve on the pulsed-wave Doppler. From the 4-chamber view, peak E-wave and A-wave velocities were acquired, and the E/A ratio was derived. Heart rate was calculated by a coupled electrocardiography.

### 2.6. *In Vitro* Myocardial Performance

Myocardial mechanic was evaluated as previously described [17, 18]. LV posterior papillary muscles were carefully dissected and vertically mounted in an organ bath heated at 29°C and filled with an oxygenated Krebs-Henseleit buffered solution 100% oxygenated and then attached to force transducer (Grass mod FT03E). Preparations were stimulated by platinum electrodes at frequency of 0.2 Hz, using square-wave pulses of 5-ms duration, and voltage adjusted to a value 10% greater than minimum required producing mechanical response. After 60 min equilibration period, the following parameters were evaluated: developed tension (DT), maximal rate of tension increase (+d*T*/d*t*) and decrease (−d*T*/d*t*), and resting tension (RT). At the end of the experiment, the muscle length was measured, and the muscular portion between the two clips was blotted dry and weighed. The cross-sectional area was estimated from the muscle weight and length by assuming a cylindrical shape and specific gravity of 1.0, and all force-related data were normalized by respective cross-sectional area and muscle mass.

### 2.7. Cardiac Biometry and Histomorphometry

Before the excision of the papillary muscle, atrium and ventricles were weighed separately, in order to use the chamber mass divided by the body mass as an index suggestive of hypertrophy. After papillary muscle dissection, ventricular samples were fixed in 10% buffered formalin and embedded in paraffin to evaluate nuclear volume and collagen content in histology slides [6, 16]. Briefly, 7 *μ*m thickness sections were obtained from the LV and hematoxylin-eosin stained. Cardiac muscle fibers were visualized on a longitudinal axis using an Olympus microscope at 40x magnification, and ellipsoid nuclei were analyzed. As an estimative of myocyte hypertrophy, the average nuclear volume was determined randomly in 50−70 myocytes cut longitudinally for each animal and calculated according to the following equation: nuclear volume = *π* × *D* × *d*2/6, where *d* is the shorter nuclear diameter and *D* is the longer nuclear diameter (15). LV collagen content was determined by picrosirius red staining using polarized light. The perivascular areas were excluded from analyzes. All histological images were visualized using an Olympus microscope at 5 randomized 40x magnification fields per animal and analyzed using Image Tool software 3.0.

### 2.8. Statistical Analysis

Analyses were performed using the SPSS (version 12.0, Chicago, Illinois, USA). All data are expressed as the mean ± standard error of the mean (SEM). The D'Agostino-Pearson and Levene tests were used to verify approximately normal statistic distributions and variance homogeneity, respectively. Comparisons between groups were performed by unpaired Student's *t* test or ANOVA two way conform necessary. Statistical significance was established at *p* < 0.05.

## 3. Results

**Figure 1:**
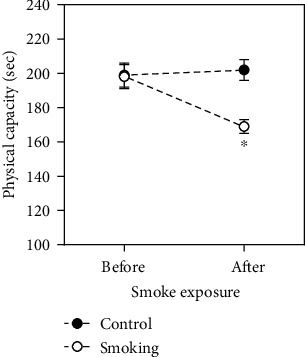
Physical capacity test performed by rats before and after smoke exposure in controls and smoking groups. Data expressed as mean + /−SEM. ^∗^*p* < 0.05 vs. before and control group.

As showed at Figure 1, no differences were found in physical capacity before interventions (baseline time-point). However, rats exposed to cigarette smoke for eight weeks exhibited patent exhaustion in a shorter time (before: 197 ± 7*vs*. after: 169 ± 4 seconds), but no changes were observed in control animals (before: 199 ± 6, after: 201 ± 5 seconds).

Despite the reduced physical capacity, no significant differences were found in systolic, diastolic, or mean blood pressures between the smoking and control groups (Table 1).

**Figure 2:**
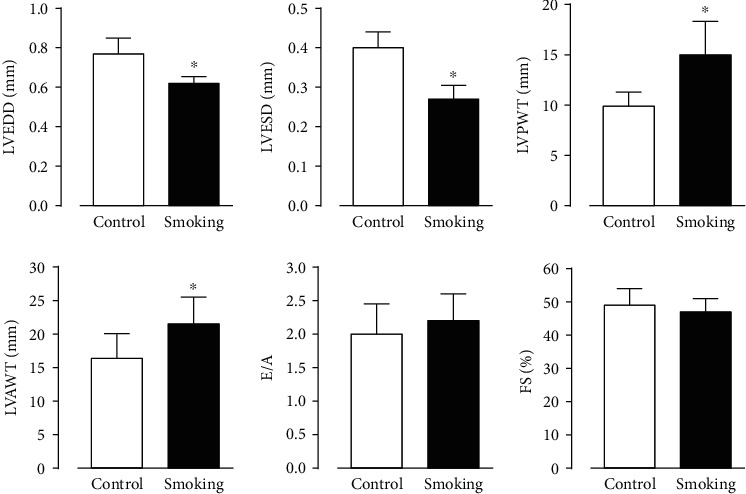
Morphofunctional data from Doppler echocardiography. Left ventricular end-diastolic (LVEDD) and end-systolic diameters (LVESD); LV posterior (LVPWT) and anterior wall thickness (LVAWT); relation between velocity of E and A waves (E/A ratio); and LV fractional shortening (FS). Bars are the means ± SEM. ^∗^*p* < 0.05*vs.* control.

Additionally, Doppler echocardiography was performed in anesthetized rats to further evaluate cardiac morphology and function. Heart rate was significantly increased in the smoking group (243 ± 28*vs.* controls: 312 ± 18 bpm), but without changes on LV fractional shortening and on the E/A ratio of the mitral valve flow study (Figure 2). Moreover, the group exposed to cigarette smoke significantly changed the cardiac morphology assessed by echocardiography: there was a reduction in LV diameters in both diastolic and systolic phases, associated with an increased LV posterior wall thickness compared with controls (Figure 2).

To further investigate if the unchanged cardiac global function *in vivo* was associated with preserved myocardial mechanics, the contractile response of papillary muscle was evaluated *in vitro*. As showed in Table 1, systolic (DT and +d*T*/d*t*) and diastolic (−d*T*/d*t*) performances of the isolated cardiac muscle were similar between groups.

Regarding renal function, as evaluated by urine protein levels and creatinine clearance, there were no statistically significant differences between the groups by short-term exposure to cigarette smoke (Table 1), although urinary protein excretion indexed by creatinine clearance tended to increase in the smoking group (*p* = 0.0552).

**Figure 3:**
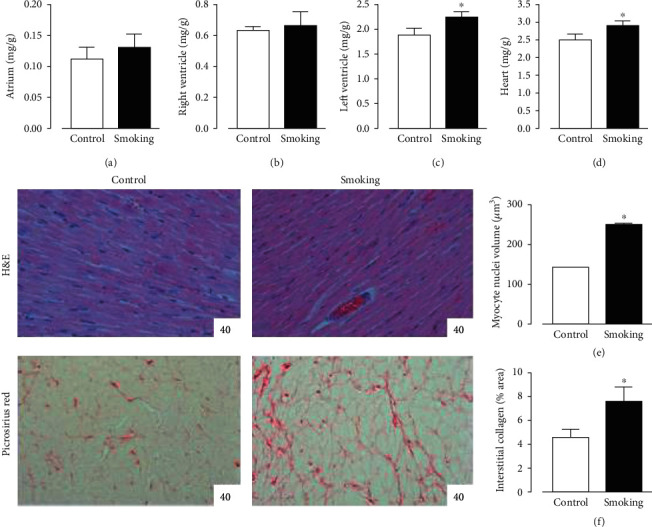
Cardiac biometry and histomorphometry. Atrial (a), right ventricular (b), left ventricular (c), and heart (d) masses were indexed to body mass. In the lower panels, representative microphotographies of the myocardium stained with hematoxylin-eosin (H&E) for myocyte nuclei analysis and picrosirius red for interstitial collagen evaluation. Remodeling parameters including myocyte hypertrophy (e) and interstitial fibrosis (f) are graphically demonstrated. Bars are the means ± SEM. ^∗^*p* < 0.05*vs.* control.

Finally, the heart biometry and histomorphometry were also assessed (Figure 3), and smoking rats had increased LV mass and total cardiac mass compared with controls. Also, microscopic study indicated an increased nuclear volume of the LV cardiomyocytes from this group. In addition, myocardial interstitial collagen content was significant increased by short-term smoke exposure.

## 4. Discussion

The present study demonstrates that short-term exposure to cigarette smoke was capable of inducing an adverse cardiac remodeling associated with impaired physical capacity in previously healthy female rats. The level of carbon monoxide in the exposure chamber used in the present study was ~510 ppm, similar to reported by other authors using the same protocol [5, 6, 19]. According to these authors, an exposure to smoke from 40 cigarettes/day is sufficient to increase the rate of carboxyhemoglobin to 5.3 ± 2.8% in rats, while animals that do not inhale smoke have 0.9 ± 0.7% of carboxyhemoglobin on blood. It should be noted that values of carbon monoxide of 400 ppm are sufficient to promote comparable to rates of carboxyhemoglobin found in humans considered heavy smokers [20]. Despite this, cardiac function evaluated in anesthetized animals and the myocardial performance evaluated *in vitro* still seems to be preserved after this short-term smoking.

Our data indicate that short-term exposure to smoke reduced the physical capacity of previously healthy rats. Actually, it is not possible to determine whether this physical capacity of smoking rats resulted from pulmonary, cardiovascular, or musculoskeletal abnormalities (i.e., skeletal muscle). However, it is noteworthy that the impairment of physical capacity is a well-known predictor of cardiovascular-related death [21] and that the improvement of the ability to perform exercise greatly contributes to reduce mortality due to cardiovascular disorders [22, 23]. Furthermore, exercise intolerance is a crucial point, because it directly affects self-related quality of life [24].

Regarding this, there are indications that chronic smoking impairs the physical capacity of the men [25, 26] and mitigates the tolerance to walking fatigue test [27]. Moreover, McDonough and Moffatt [28] stated that the chronically increased smoking-related blood carbon monoxide content could worsen exercise tolerance and reduce the maximal aerobic capacity, associated with an impaired glucose metabolism during exercise. An experimental study has also shown a reduction of 24% of aerobic capacity of animals exposed to carbon monoxide in similar dose to that found in individuals who smoke heavily [29]. For the authors, abnormalities in mitochondrial function in skeletal muscles can interfere with exercise capacity.

To the best of our knowledge, the impact of short-term cigarette smoking on the functional fitness and physical activity is few related by the literature. Regarding the effects of cigarette smoke on the cardiac function as a substrate for impaired physical capacity, there was proposed a possible association between smoking and functional fitness in heart failure [30]. However, given that both chronic smoking and heart failure syndrome could potentially reduce physical capacity not only by cardiac but also by peripheral changes such as pulmonary, hemodynamic, and neuro-motor functions, associated with water and salt kidney retention, our data indicates that short-term cigarette smoking may reduce functional fitness independent of heart or kidney failures.

Actually, there were no significant changes on GFR and urinary protein excretion in rats submitted to passive cigarette smoking for eight weeks, suggesting preserved glomerular and tubular functions. On the other hand, there are evidences that chronic cigarette smoking is associated with functional and structural renal changes in rats, demonstrating abnormal glomerular morphology after 16 weeks [11] and advanced hydropic degeneration of kidney after 24 weeks [31].

Conversely to previous observed in chronic smoke exposure [5, 6], but similar to described for short-term protocols [8, 9], echocardiographic data did not show ventricular systolic or diastolic dysfunction after this 8-week protocol, and no changes on blood pressure were noted as well. Moreover, cardiac muscle mechanics assessed *in vitro* was not affected, similar to also reported by Paiva et al. [19] after 4 weeks and Brooks et al. [32] after 16 weeks of smoking exposure in rats. Together, these findings could suggest that our experimental model of a short-term cigarette smoke exposure was not accompanied by cardiac dysfunction. However, about this apparent normality it is important noting that, under anesthesia or without substantial hemodynamic/mechanical loads, cardiac performance may appear unaltered, making the appropriate characterization of dysfunction difficult [15]. In spite of this supposed normality, when the hearts of smoking rats were submitted to a stress condition such as the physical capacity swim test, a significant degree of impairment was evident. Although we did not perform hemodynamic stress test and considering that noncardiac effects of smoking may also be present, we could just hypothesized that impaired physical capacity was due, at least in part, to a reduced cardiac reserve.

Notwithstanding, a previous study demonstrated that short-term of cigarette smoke induced cardiac morphological changes in rats [6] that could also be related to the reduced physical capacity. In fact, the increase in heart mass and myocyte nuclear volume and the reduced ventricular diameter after short-term cigarette smoking as suggestive of cardiac hypertrophy are not new data. There is no consensus about the hypertrophy mechanism involved in cardiac hypertrophy; however, the hypoxia caused carbon monoxide and nicotine. In the present study, the hypertrophic and fibrotic processes were not associated with changes on blood pressure, which suggests that this cardiac remodeling may be independent of hemodynamic loading. Regarding this, an important issue is the increased blood viscosity that could sustain increased cardiac afterload. Moreover, several other factors could act as additional mechanisms to trigger cardiac hypertrophic process during cigarette smoking: the generation of reactive oxygen species induced by smoking is knowingly cytotoxic to the myocardium [33]; nicotine has also been shown to be associated with myocardial remodeling [34, 35] and sympathetic hyperactivity [7], and although we did not assess sympathetic tone, heart rate was significantly elevated in the smoking group, which could play a role for the adverse cardiac remodeling; and evidence for the association between carbon monoxide/hypoxia and myocardial hypertrophy had also been showed [29, 36]. Although we did not measure plasma levels of nicotine and cotinine, it is important note that these parameters are commonly used to estimate smoking intensity. In the present work, we used the carbon monoxide content on the exposure chamber to estimate smoking intensity, similar to other studies [5, 6, 19]. Thus, it is possible to consider that the hypertrophic response was linked to multiple effects of cigarette smoking not only to nervous and cardiovascular systems, but also by neurohumoral mechanisms. For example, it is well known that smoking, or more specifically nicotine, activates the sympathoadrenal system and increases the synthesis and release of noradrenaline and adrenaline [7, 37, 38], and stimulates the renin-angiotensin-aldosterone system [39].

In conclusion, our results indicate that short-term cigarette smoke exposure in rats induced a concentric LV hypertrophy accompanied by increased in interstitial collagen. In addition, despite the apparent normal cardiac and renal functions, the smoking group has a significant impairment of the physical capacity, which could be suggested a result, at least in part, of a reduced cardiac reserve as a damaging consequence of this short-term smoking. It is noteworthy that recurrence of short-term smoking may have summative effects and thus constitute the mechanism underlying the ultimate chronic effects. Therefore, our data about the short-term effect of cigarette smoking can reflect an initial change in the pathophysiological mechanisms of smoking-induced chronic harmfulness.

## Figures and Tables

**Table 1:** Parameters of cardiac and renal functions of animals not exposed and exposed to cigarette smoke.

	Control	Smoking	*p* value
Hemodynamic			
SBP (mmHg)	126 ± 2	129 ± 2	*p* > 0.05
DBP (mmHg)	84 ± 2	82 ± 3	*p* > 0.05
MBP (mmHg)	98 ± 1	98 ± 2	*p* > 0.05
Myocardial function			
DT (g/mm^2^/mg)	1.11 ± 0.57	0.95 ± 0.38	*p* > 0.05
+d*T*/d*t*_max_ (g/mm^2^/mg/s)	10.13 ± 3.57	8.63 ± 2.62	*p* > 0.05
−d*T*/d*t*_max_ (g/mm^2^/mg/s)	−8.18 ± 5.88	−5.43 ± 1.82	*p* > 0.05
RT (g/mm^2^/mg)	0.22 ± 0.17	0.19 ± 0.06	*p* > 0.05
Renal function			
SCr (mg/dL)	0.48 ± 0.05	0.47 ± 0.08	*p* > 0.05
CrCl (mL/min)	1.14 ± 0.02	1.13 ± 0.05	*p* > 0.05
Uprot (mg/24 h)	16.27 ± 1.60	20.15 ± 1.53	*p* > 0.05
Uprot/CrCl (mg/mL)	14.78 ± 1.40	18.56 ± 1.20	*p* > 0.05

Values are the mean ± SEM. Systolic (SBP), diastolic (DBP), and mean blood pressures (MBP) measured in hemodynamic study; developed (DT) and resting tensions (RT) and maximum rate of tension rise (+d*T*/d*t*_max_) and decline (-d*T*/d*t*_max_) of isolated cardiac muscles; serum creatinine (SCr), creatinine clearance (ClCr), 24 h urinary excretion (Uprot), and urine protein/creatinine clearance ratio (Uprot/CrCl).

## Data Availability

All data are available if necessary.
